# Magnetic-controlled membrane transport

**DOI:** 10.1093/nsr/nwab062

**Published:** 2021-04-08

**Authors:** Rob G H Lammertink

**Affiliations:** Soft matter, Fluidics and Interfaces, Faculty of Science and Technology/Mesa+ Institute for Nanotechnology, University of Twente, The Netherlands

Liquid infused membranes form an innovative class of liquid infused structures presenting highly interesting features, the full potential of which we are only beginning to appreciate. The key components include a porous host that is infused with a liquid that has a high affinity for this host, resulting in strong conformal wetting of the inner porous structure. The transport of another immiscible phase through these liquid infused structures is evidently governed by the characteristics of the infusion liquid and the porous host.

During such permeating transport, these membranes present a lubricating fluid layer on the pore surface, with sufficient interaction strength to maintain this pore lubrication during permeation. Such liquid-lined geometries provide a vast variety of interesting aspects, including the interfacial tension gating of phases [1], for example liquid-gas or liquid-liquid separations, and reduced biofouling [2].

The performance of liquid infused membranes typically relies on the capillary force that stabilizes the infused liquid, resulting in pore opening upon exceeding the Laplace pressure [3]. Such a mechanism is governed by the interfacial tension between the infused liquid and invading phase, setting the threshold pressure for permeation. With this mechanism, interesting separations have been demonstrated between immiscible phases. One highly interesting aspect is the role of the lubrication layer during the invading phase permeation. Evidently, the stress exerted by the invading fluid is directly influenced by the infusion liquid characteristics and its lining thickness and confinement.

Recently, the group of Hou [4] described magnetorheological infusion liquids for liquid gating purposes. This innovation provides additional control of the infusion liquid characteristics and thereby of the resulting membrane function. The porous media, copper foam, was infused by this magnetorheological fluid, giving rise to externally tunable permeability. The orientation and alignment of the colloidal magnetic particles of the infusion liquid provides anisotropy to the rheological properties. Hence, the fluid response to an invading liquid depends on the magnitude and direction of an externally applied magnetic field. The mechanism of such permeability control is fundamentally different from the previously reported capillary pressure-controlled gating. Using a magnetorheological fluid, its confinement in the pore and external applied magnetic field affects the displacement by an invading liquid based on the rheological characteristics. Experiments indicated a strong correlation between the threshold pressure for fluid invasion and the magnetic field strength, for which a model was constructed. The rheological anisotropy was further analyzed based on entropy calculations of strongly confined magnetic colloids. A general applicability of this approach is expected for other externally controlled colloidal systems based on acoustics, light and electric fields.

***Conflict of interest statement*. ** None declared.
